# The Spatiotemporal Evolution of MRI-Derived Oxygen Extraction Fraction and Perfusion in Ischemic Stroke

**DOI:** 10.3389/fnins.2021.716031

**Published:** 2021-08-16

**Authors:** Di Wu, Yiran Zhou, Junghun Cho, Nanxi Shen, Shihui Li, Yuanyuan Qin, Guiling Zhang, Su Yan, Yan Xie, Shun Zhang, Wenzhen Zhu, Yi Wang

**Affiliations:** ^1^Department of Radiology, Tongji Hospital, Tongji Medical College, Huazhong University of Science and Technology, Wuhan, China; ^2^Department of Radiology, Weill Cornell Medicine, New York, NY, United States; ^3^Department of Biomedical Engineering, Cornell University, Ithaca, NY, United States

**Keywords:** cerebral metabolic rate of oxygen, DWI reversal, ischemic stroke, ischemic penumbra, magnetic resonace imaging, oxygen extraction fraction, quantitative susceptibility mapping

## Abstract

**Purpose:**

This study aimed to assess the spatiotemporal evolution of oxygen extraction fraction (OEF) in ischemic stroke with a newly developed cluster analysis of time evolution (CAT) for a combined quantitative susceptibility mapping and quantitative blood oxygen level-dependent model (QSM + qBOLD, QQ).

**Method:**

One hundred and fifteen patients in different ischemic stroke phases were retrospectively collected for measurement of OEF of the infarcted area defined on diffusion-weighted imaging (DWI). Clinical severity was assessed using the National Institutes of Health Stroke Scale (NIHSS). Of the 115 patients, 11 underwent two longitudinal MRI scans, namely, three-dimensional (3D) multi-echo gradient recalled echo (mGRE) and 3D pseudo-continuous arterial spin labeling (pCASL), to evaluate the reversal region (RR) of the initial diffusion lesion (IDL) that did not overlap with the final infarct (FI). The temporal evolution of OEF and the cerebral blood flow (CBF) in the IDL, the RR, and the FI were assessed.

**Results:**

Compared to the contralateral mirror area, the OEF of the infarcted region was decreased regardless of stroke phases (*p* < 0.05) and showed a declining tendency from the acute to the chronic phase (*p* = 0.022). Five of the 11 patients with longitudinal scans showed reversal of the IDL. Relative oxygen extraction fraction (rOEF, compared to the contralateral mirror area) of the RR increased from the first to the second MRI (*p* = 0.044). CBF was about 1.5-fold higher in the IDL than in the contralateral mirror area in the first MRI. Two patients showed penumbra according to the enlarged FI volume. The rOEF of the penumbra fluctuated around 1.0 at earlier scan times and then decreased, while the CBF decreased continuously.

**Conclusion:**

The spatiotemporal evolution of OEF and perfusion in ischemic lesions is heterogeneous, and the CAT-based QQ method is feasible to capture cerebral oxygen metabolic information.

## Introduction

Ischemic stroke is one of the leading causes of disability and mortality globally (Kim et al., 2020). Advanced imaging techniques that provide diversity of ischemic tissue characteristics from hemodynamics (Hernandez-Perez et al., 2016) to metabolic biomarkers (An et al., 2015; Harston et al., 2015; Barca et al., 2020) have enabled better pathophysiological understanding of ischemic stroke and better therapeutics.

The oxygen extraction fraction (OEF) is the relative difference of the oxygen concentration between arterial and venous blood, a practical parameter representing the oxygen consumption of brain tissue. It plays a vital role in sustaining the normal coupling between cerebral blood flow (CBF) and the cerebral metabolic rate of oxygen (CMRO_2_), as expressed by the following equation:

$${\text{CMRO}}_{2}=\text{OEF}\cdot \text{CBF}\cdot {\text{[H]}}_{\text{a}}$$

where [H]_a_ is the oxygenated heme molar concentration in the arteriole (Zhang et al., 2018). Changes in OEF reflect different tissue viabilities and cerebral neurometabolic states. Positron emission tomography (PET) has so far been used as the gold standard for cerebral OEF mapping (Baron, 1985; Sette et al., 1989; Kudo et al., 2016), but radiation exposure is unavoidable and it consumes a large amount of expensive materials. As a result, substantial studies (Stout et al., 2018; Cherukara et al., 2019; Hubertus et al., 2019; O’Brien et al., 2019; Ma et al., 2020) have emerged in the field of magnetic resonance imaging using either phase or magnitude information for OEF measurement in cerebrovascular diseases (Uwano et al., 2017; Kato et al., 2018; Stone et al., 2019), neurodegenerative disorder (Lin et al., 2019), and other systemic diseases (Fields et al., 2018; Miyata et al., 2019). Advancement in imaging technology is of great benefit to the diagnosis and therapeutic strategy making for ischemic stroke; however, the oxygenation status of an ischemic brain tissue has a broad range depending on the blood supply and the time from stroke onset, and there are few studies systematically reporting the oxygenation status through different time points and blood flow.

Diffusion-weighted imaging (DWI) can show ischemic lesions within minutes from stroke onset and has been considered to represent the tissue that is irreversibly damaged. Nevertheless, studies have reported that some diffusion lesions are reversible if the infarct volume is small (Asdaghi et al., 2014) or with endovascular treatment (Inoue et al., 2014). These findings are mostly based on the morphology, while the metabolic information of the reversal region (RR) is still unexplored.

Given this, we aimed to assess the spatiotemporal evolution of OEF in ischemic lesions among different stroke phases and to identify the metabolic characteristics of the tissue reversed on DWI using a novel MRI-based vascular challenge-free OEF mapping method developed by Cho et al. (2020b) for ischemic stroke patients. This method is based on three-dimensional (3D) multi-echo gradient recalled echo (mGRE) data and combines quantitative susceptibility mapping and quantitative blood oxygen level-dependent model (QSM + qBOLD, QQ) with cluster analysis of time evolution (CAT-based QQ thereafter).

## Materials and Methods

### Patient Population

This retrospective study was approved by the ethics committee of the local institution, which waived written informed consent from subjects.

One hundred and thirty-seven patients with ischemic stroke from January 2014 to December 2019 were retrospectively collected from our institution using the following inclusion criteria: (1) being diagnosed with first ever unilateral ischemic stroke; (2) receiving MRI scans including 3D mGRE and conventional sequences; and (3) with known symptom onset time. The exclusion criteria included other neurological or systemic diseases or apparent motion artifact in MRI data. Twenty-two patients were excluded due to brain stem or cerebellum infarction (*n* = 8), rather small lesion volume (<250 mm^3^, *n* = 2), hemorrhage transformation shown on reconstructed QSM maps (*n* = 4), and thrombolytic or endovascular thrombectomy therapy before MRI examination (*n* = 8).

Thus, 115 patients remained in the final analysis and were divided into four groups according to the time interval from stroke symptom onset to MRI scan (Fung et al., 2011): (1) acute phase (≤1 day, *n* = 14); (2) early subacute phase (1–7 days, *n* = 54); (3) late subacute phase (8–14 days, *n* = 22); and (4) chronic phase (>14 days, *n* = 25). Among these patients, 11 received two longitudinal MRI scans. The first MRIs were performed on admission within the early subacute phase and the second were on days 8–40 after the onset of symptoms.

The clinical severity was evaluated using the National Institutes of Health Stroke Scale (NIHSS) by an experienced neurologist (8 years) right before the MRI scan.

### Imaging Acquisition and Processing

All MRI examinations were conducted on a 3-T MRI system using a 32-channel head coil (Discovery MR750, GE Healthcare, Milwaukee, WI, United States). The imaging protocol was composed of 3D mGRE, structural 3D T1-weighted imaging (3D-T1WI), T2 fluid-attenuated inversion recovery (T2FLAIR), and DWI. The acquisition parameters for 3D mGRE were: field of view (FOV) = 24 cm, repetition time (TR) = 42.8 ms, TE1/ΔTE = 4.5/4.9 ms (time to echo, TE), number of TEs = 8, acquisition matrix = 416 × 320, readout bandwidth = 244 Hz/pixel, slice thickness = 2 mm, flip angle = 20°C, and number of averages = 1. 3D-T1WI was acquired using brain volume (BRAVO) sequence with TR/TE/TI = 7.1/2.7/450 ms, flip angle = 12°C, matrix = 256 × 256, FOV = 240 × 280 mm^2^, number of averages = 1, slice thickness = 1mm, and number of slices = 184.

Quantitative susceptibility mapping was reconstructed from 3D mGRE data using a fully automated zero-referenced morphology enabled dipole inversion (MEDI+0) method that uses the ventricular cerebrospinal fluid (CSF) as a zero reference (Liu et al., 2018). OEF maps were calculated based on 3D mGRE data using the CAT-based QQ model, following the steps below as in the referred literature (Cho et al., 2020b).

QQ is formulated as

$$Y^{*},\nu^{*},R_{2}^{*},S_{0}^{*},\chi_{\text{nb}}^{*}={\text{argmin}}_{Y,\nu ,R_{2},S_{0},\chi_{\text{nb}}}\left\{\begin{array}{c} w{||F_{\text{QSM}}\left(Y,\nu ,\chi_{\text{nb}}\right)-\chi ||}_{2}^{2}\\ +{||S\left(t\right)-S_{\text{qBOLD}}\left(S_{0},Y,\nu ,R_{2},\chi_{\text{nb}},t\right)||}_{2}^{2}\\ +\lambda {\left(\bar{\text{OEF}\left(Y\right)}-{\text{OEF}}_{\text{wb}}\right)}^{2} \end{array}\right\}$$

where *Y* is the venous oxygenation that can be converted into OEF (OEF = 1−*Y*/*Y*_a_, with arteriole oxygenation *Y*_*a*_ = 0.98) (Cho et al., 2018), ν is venous blood volume, χ_nb_ is the susceptibility of non-blood materials in the tissue, *w* is the weighting on the QSM term, and

$$F_{\text{QSM}}\left(Y,\nu ,\chi_{\text{nb}}\right)=\left[\frac{\chi_{\text{ba}}}{\alpha }+\Psi_{\text{Hb}}\cdot \Delta_{\chi }\text{Hb}\cdot \left(-\text{Y}+\frac{1-\left(1-\alpha \right)\cdot Y_{\text{a}}}{\alpha }\right)\right]\cdot \nu +\left(1-\frac{\nu }{\alpha }\right)\cdot \chi_{\text{nb}}$$

with χ as the measured susceptibility, χ_ba_ the fully oxygenated blood susceptibility, α = 0.77 the ratio between the venous and total blood volume, Ψ_Hb_ = 0.0909 the hemoglobin volume fraction assuming Hct = 0.357, and Δ_χHb_ = 12,522 ppb as the susceptibility difference between deoxy- and oxyhemoglobin.

*S*_qBOLD_(*t*) represents the qBOLD method modeling the mGRE magnitude in a voxel:

$$S_{\text{qBOLD}}\left(t\right)=S_{0}\cdot e^{-R_{2}\cdot t}\cdot F_{\text{BOLD}}\left(Y,\nu ,\chi_{\text{nb}},t\right)\cdot G\left(t\right)$$

where *G*(*t*) is the macroscopic field effect at time *t* determined *via* the voxel spread function [see Appendix in Cho et al. (2018)] and *F*_*BOLD*_ is the deoxygenated blood effect inside the voxel.

A physiological constraint was imposed in that the whole brain OEF average, $\bar{\text{OEF}\left(Y\right)}$, should be close to the OEF estimation from a main draining vein, the straight sinus, OEF_wb_ = Hct_vt_⋅OEF_ss_, where Hct_vt_ = 0.75 is the hematocrit ratio between large vessels and the brain tissue (Sakai et al., 1985) and OEF_ss_ = 1−*Y*_ss_/*Y*_a_, with *Y*_*ss*_ estimated by inserting the average straight sinus susceptibility into *F*_*QSM*_ with Ψ_Hb_ = 0.1197 (Savicki et al., 1984; Hoffman et al., 2017), ν = 1, and χ_nb_ = 0.

For robust QQ-based OEF estimation via effective signal-to-noise ratio (SNR) improvement, CAT was used. Voxels with similar signal decays, *S*_qBOLD_(*t*)/*G*(*t*), are grouped into a cluster and assumed to have similar tissue parameter values (*Y*, ν, and *R*_2_). For clustering, CAT used X-means, a modified K-means algorithm that automatically selects the optimal number of clusters based on the Bayesian information criterion (Dau Pelleg, 2000).

For the 11 patients in the longitudinal scans, 3D pseudo-continuous arterial spin labeling (pCASL; TR/TE/label time/post-label delay = 4,787/14.6/1,500/1,525 ms, FOV = 24 cm, slice thickness = 4 mm, 34 control pairs) was simultaneously acquired using an interleaved 3D stack of spirals fast spin echo (FSE) sequence with a high-level background suppression. CBF (in milliliters per 100 g/min) maps were generated on the GE workstation (Advanced Workstation 4.6, GE Medical Systems) using a kinetic model proposed by Alsop and Detre (1996).

### Image Analysis

All images within a single time point were co-registered and interpolated to the resolution of the QSM maps using the FSL FLIRT algorithm (Jenkinson et al., 2002). For the 115 patients, the regions of interest (ROIs) of the infarcted area were drawn on the co-registered DWI and apparent diffusion coefficient (ADC) images using the ITK-SNAP software (version 3.8.0^1^) by an experienced neuroradiologist (5 years) who was blind to the group allocation. The ROIs were then overlaid to the co-registered OEF maps.

**FIGURE 1:**
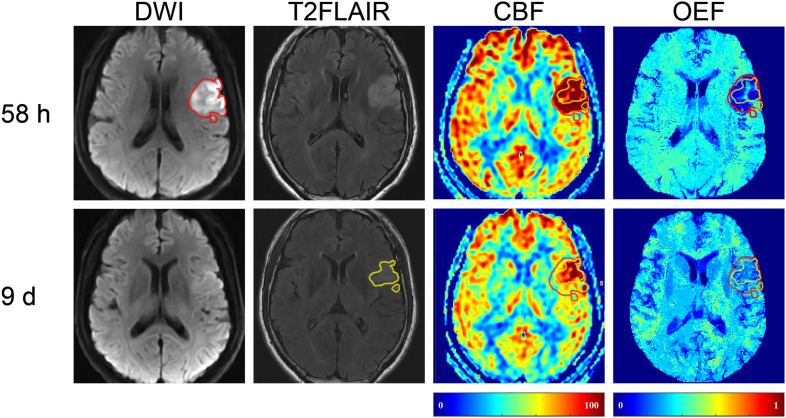
Representative images of a 57-year-old male with right limb weakness. The final infarct (FI, with a volume of 8.3 ml on 9-day T2 fluid-attenuated inversion recovery (T2FLAIR) (shown in yellow) was smaller than the 58-h initial diffusion lesion (IDL, 13.18 ml) on diffusion-weighted imaging (DWI) (shown in red). The area of IDL that did not overlap with FI was the reversal region (RR). DWI, diffusion-weighted imaging; T2FLAIR, T2 fluid-attenuated inversion recovery; CBF, cerebral blood flow; OEF, oxygen extraction fraction.

For the 11 patients with longitudinal scans, image registration was performed using nonlinear registration of the T1-weighted structural scans. We defined hyperintensity on the first DWI as the initial diffusion lesion (IDL), hyperintensity on the second T2FLAIR as the final infarct (FI), and the area of IDL that did not overlap with the FI as the RR, if there is any (Figure 1). The ROIs were copied on to the OEF and CBF maps from all the other MRI scans to characterize the respective temporal changes.

The OEF values of the ROIs were extracted and the relative oxygen extraction fraction (rOEF) was calculated by dividing the OEF values of the ROIs with those of the mirrored contralateral side. So were the CBF values. Voxels close to or beyond the brain edge were carefully excluded to avoid potential confounds of non-brain regions.

### Statistical Analysis

All data were analyzed using IBM SPSS Statistics 26 (Armonk, NY, United States). Kruskal–Wallis test and chi-square test were used for group comparisons of the demographics. The datasets of OEF, rOEF, CBF, and relative cerebral blood flow (rCBF) used in the following analysis all complied with normal distribution (Kolmogorov–Smirnov test) and homogeneity of variance (Levene’s test). We used two-tailed paired *t*-test and ANOVA with *post hoc* Bonferroni test for the comparisons of OEF, rOEF, CBF, and rCBF between different the stroke phases and the different ROIs. The correlations between OEF and clinical severity (NIHSS) were assessed using partial correlation analysis with the confounding factor (lesion volume) corrected. *P* < 0.05 was recognized as statistically significant.

## Results

### Cross-Sectional Comparisons of OEF and rOEF

The detailed demographic information of the 115 patients is provided in Table 1.

**TABLE 1:** Group demographics and imaging characteristics of the 115 included patients.

	Acute (≤1 day)	Early subacute (1–7 days)	Late subacute (8–14 days)	Chronic (>14 days)	*p^a^*
	*n* = 14	*n* = 54	*n* = 22	*n* = 25	
**Clinical characteristics**					
Age (years), mean ± SD	55.0 ± 8.2	57.4 ± 12.5	54.1 ± 10.5	54.5 ± 9.9	0.602
Sex, male (%)	13(93)	40(74)	18(82)	16(64)	0.202
**Vascular risk factors**					
Hypertension, *n* (%)	9(64)	43(80)	18(82)	15(60)	0.186
Diabetes mellitus, *n* (%)	4(29)	12(22)	7(32)	2(8)	0.214
Dyslipidemia, *n* (%)	4(29)	12(22)	2(9)	0(0)	0.031*
Heart disease, *n* (%)	2(14)	7(13)	2(9)	0(0)	0.292
Smoking, *n* (%)	12(86)	26(48)	11(50)	12(48)	0.077
Alcohol, *n* (%)	8(57)	26(48)	11(50)	9(36)	0.593
NIHSS, median (IQR)	6(4–12)	5.5(3–8)	4(2–8)	2(1–4.5)	0.006*
Time to MRI scan (days), median (IQR)	0.8(0.5–1.0)	3.5(2.5–5.5)	10.5(9.0–11.9)	30.0(20.0–30.0)	<0.0001*
Lesion volume (ml), median (IQR)	8.8(4.3–47.8)	3.9(1.1–19.8)	8.4(2.8–14.0)	10.4(4.5–21.1)	0.17
OEF of the infarcted area, mean ± SD(%)	26.04 ± 7.24	21.92 ± 3.86	21.87 ± 4.07	21.58 ± 5.04	0.022*
rOEF, mean ± SD	0.85 ± 0.14	0.73 ± 0.13	0.73 ± 0.12	0.75 ± 0.13	0.024*

NIHSS, National Institutes of Health Stroke Scale; OEF, oxygen extraction fraction; rOEF, relative oxygen extraction fraction; IQR, interquartile range. *p < 0.05 (statistically significant). ^*a*^Difference among four groups.

**FIGURE 2:**
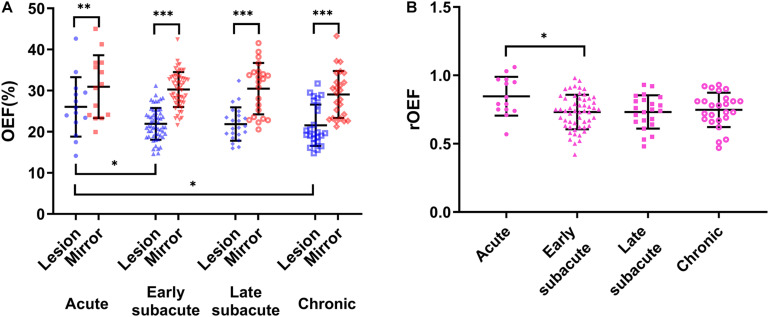
Oxygen extraction fraction (OEF) of the infarcted area defined on DWI and the contralateral mirror area and their quotient [relative OEF, relative oxygen extraction fraction (rOEF)] in four ischemic stroke phases. **(A)** The OEF significantly reduced compared with the contralateral mirror area in all stroke phases (two-tailed paired *t*-test: *p* < 0.05 for all). Besides, the OEF showed a trend of decrease from the acute to the chronic phase and was statistically significant (ANOVA: *p* = 0.022). **(B)** The rOEF showed the same decreasing tendency with OEF (ANOVA: *p* = 0.024). The center line is the mean and the other two lines are the standard deviations. *p < 0.05, **p < 0.01, and ***p < 0.001.

The OEF of the infarcted area identified on DWI was significantly lower than that of the contralateral mirror area regardless of stroke phases (acute phase: infarcted area 26.04 ± 7.24% vs. mirror 30.95 ± 7.65%, *p* = 0.001; early subacute phase: 21.92 ± 3.86% vs. 30.27 ± 4.25%, *p* < 0.001; late subacute phase: 21.87 ± 4.07% vs. 30.48 ± 6.23%, *p* < 0.001; chronic phase: 21.58 ± 5.04% vs. 29.06 ± 5.72%, *p* < 0.001). Besides, both the OEF and rOEF of the infarcted area showed statistical differences among the four stroke phases (*p* = 0.022 and 0.024, respectively), with a trend of decline from the acute to the chronic phase (Figure 2).

Both the OEF and the NIHSS score decreased from the acute to the chronic phase. However, the correlations between them did not reach statistical significance. When analyzed in the four stroke phases separately, the NIHSS score positively correlated with OEF only in the acute phase (*r* = 0.654, *p* = 0.021).

### Longitudinal Comparisons of OEF and rOEF

The clinical characteristics of the 11 patients with longitudinal MRI scans are provided in Supplementary Table 1.

**FIGURE 3:**
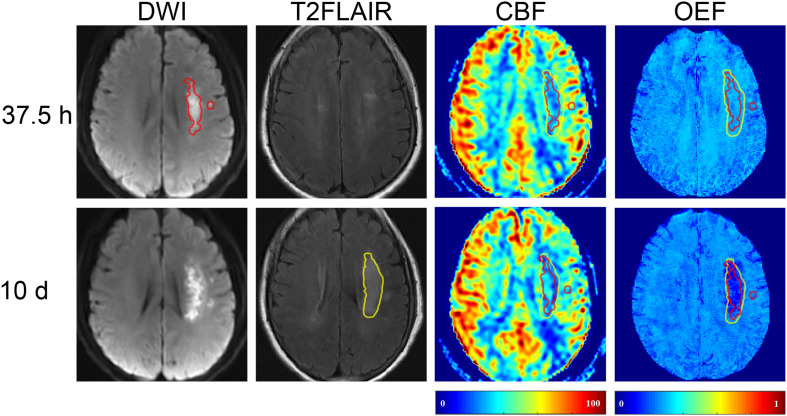
Representative images of a 64-year-old female with right limb weakness and aphasia. The FI (with a volume of 5.31 ml) on 10-day T2FLAIR (shown in yellow) was larger than the 37.5-h IDL (10.97 ml) on DWI (shown in red). The area of FI that did not overlap with IDL was the mismatch. T2FLAIR, T2 fluid-attenuated inversion recovery; DWI, diffusion-weighted imaging.

**FIGURE 4:**
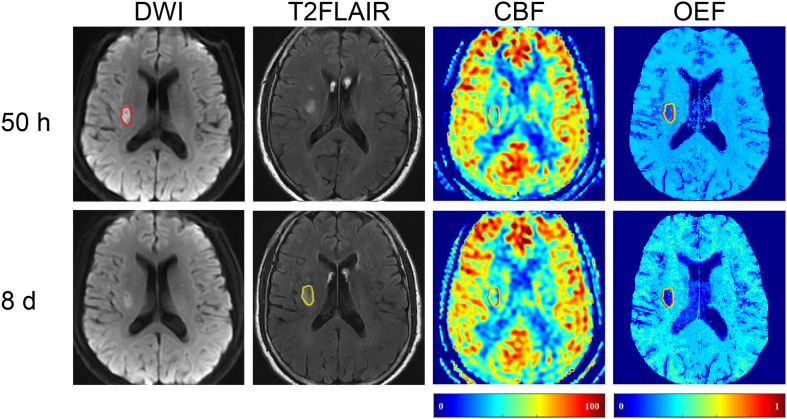
Representative images of a 59-year-old male with left limb weakness. The FI (with a volume of 2.82 ml) on 8-day T2FLAIR (shown in yellow) was equal to the 50-h IDL (2.82 ml) on DWI (shown in red). T2FLAIR, T2 fluid-attenuated inversion recovery; DWI, diffusion-weighted imaging.

In five (45.5%) of the 11 patients (cases 3, 4, 8, 9, and 10), the volume of FI was found to be smaller than that of the IDL (Figure 1), indicating a regional diffusion reversal (DR group). The IDLs were located in the frontal cortex (*n* = 1), temporal cortex (*n* = 2), and the temporal cortex and basal ganglia (*n* = 2). The other six patients had either enlarged (cases 6 and 11; Figure 3) or equal (cases 1, 2, 5, and 7; Figure 4) FI volumes to the IDL without diffusion reversal (NDR group), with the IDLs located in the corona radiata and basal ganglia (*n* = 4) and in the corona radiata and centrum semiovale (*n* = 2).

Compared with the NDR group, the DR group had larger IDLs and FI volumes but gentler clinical symptoms, although the difference did not reach statistical significance. In the first MRI, the CBF and rCBF of the IDLs of the DR group were higher than those of the NDR group (*p* = 0.005 and 0.023, respectively), while the OEF and rOEF between the two groups were not significantly different (*p* = 0.481 and 0.218, respectively). In the second MRI, the rOEF of the FI of the DR group were higher than those of the NDR group (*p* = 0.002). The rOEF of the NDR group decreased from the first to the second MRI (*p* = 0.006), while that of the DR group did not (*p* = 0.252). Detailed comparisons between the two groups are shown in Table 2.

**TABLE 2:** Characteristics of patients with and without diffusion reversal.

	DR (*n* = 5)	NDR (*n* = 6)	*p*
Age (years), mean ± SD	45.4 ± 11.3	56.3 ± 4.7	0.097
Sex, male (%)	4(80.0)	5(83.3)	0.887
**First MRI, IDL**			
NIHSS, median (IQR)	2(1.5–6)	5.5(2–8)	0.329
TI (h) mean ± SD	73.70 ± 16.92	55.25 ± 16.44	0.101
Volume (mm^3^), median (IQR)	13.18(1.91–32.77)	4.07(2.26–6.22)	0.171
OEF (%), mean ± SD	20.27 ± 2.52	22.18 ± 5.30	0.481
rOEF, mean ± SD	0.70 ± 0.17	0.82 ± 0.13^a^	0.218
CBF (ml100 g^–1^min^–1^), mean ± SD	85.43 ± 23.73	30.89 ± 6.79	0.005*
rCBF, mean ± SD	1.45 ± 0.26	0.95 ± 0.33	0.023*
**Second MRI, FI**			
NIHSS, median (IQR)	1(0.5–1.5)	5(1–7.25)	0.082
TI (days), mean ± SD	17.80 ± 13.27	10.50 ± 1.76	0.287
Volume (mm^3^), median (IQR)	8.30(0.91–13.31)	5.00(2.11–13.47)	0.950
OEF (%), mean ± SD	22.23 ± 3.36	20.38 ± 4.50	0.470
rOEF, mean ± SD	0.79 ± 0.05	0.64 ± 0.06	0.002*
CBF (ml 100 g^–1^min^–1^), mean ± SD	48.40 ± 30.29	34.81 ± 16.53	0.367
rCBF, mean ± SD	0.99 ± 0.46	0.99 ± 0.36	0.990

DR, group with diffusion reversal; NDR, group without diffusion reversal; IDL, initial diffusion lesion; TI, time interval from stroke symptom onset to first/second MRI; CBF, cerebral blood flow; rCBF, relative cerebral blood flow; FI, final infarct; IQR, interquartile range. *p < 0.05 (statistically significant). ^*a*^rOEF of the IDL in the first MRI was significantly different from that of the FI in the second MRI (*p* = 0.006).

**FIGURE 5:**
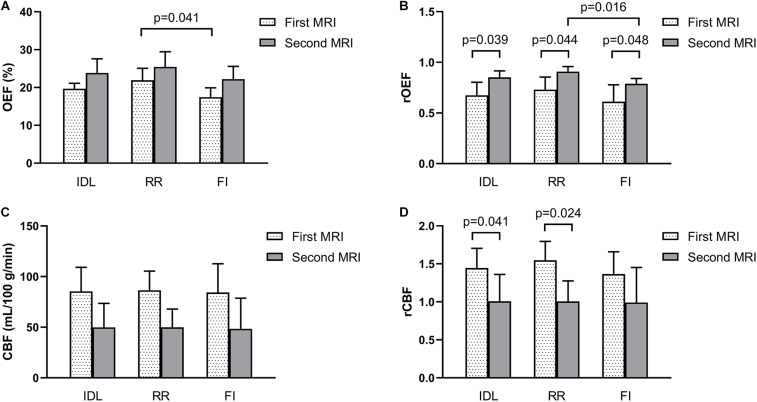
Parameters of the IDL, RR, and FI between the longitudinal MRI scans in five patients who showed reversal of IDL. **(A)** OEF. **(B)** rOEF. **(C)** CBF. **(D)** rCBF. OEF, oxygen extraction fraction; rOEF, relative OEF; CBF, cerebral blood flow; rCBF, relative CBF; IDL, initial diffusion lesion; RR, reversal region; FI, final infarct.

In patients with regional reversal of restricted diffusion lesion, the rOEF of the IDL, RR, and FI all increased from the first MRI (0.67 ± 0.13, 0.73 ± 0.12, and 0.61 ± 0.17) to the second MRI (0.85 ± 0.06, 0.90 ± 0.05, and 0.79 ± 0.05; *p* = 0.039, 0.044, and 0.048, respectively) (Figure 5B). Although the difference in the OEF was not significant, there was a trend of increase with time. The OEF of the RR (21.93 ± 3.14) was higher than that of the FI (17.45 ± 2.46) in the first MRI (*p* = 0.041; Figure 5A), and the rOEF of the RR was higher than that of the FI in the second MRI (*p* = 0.016; Figure 5B). At the first MRI scan, the lesions were hyperperfused, with the CBF significantly higher than the contralateral mirror area, while in the second scan, the CBF decreased to or below the normal level (Figures 5C,D).

**FIGURE 6:**
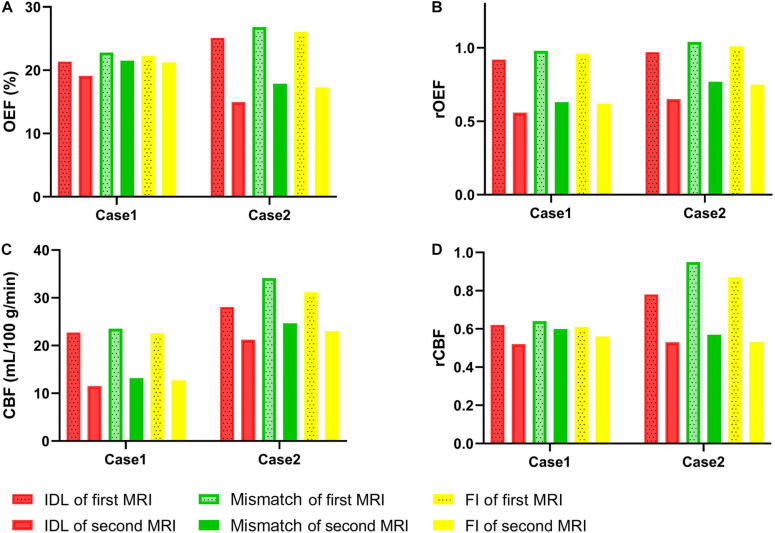
Parameters of IDL, mismatch, and FI between the longitudinal MRI scans in two patients with enlarged lesion volume. **(A)** OEF. **(B)** rOEF. **(C)** CBF. **(D)** rCBF. OEF, oxygen extraction fraction; rOEF, relative OEF; CBF, cerebral blood flow; rCBF, relative CBF; IDL, initial diffusion lesion; FI, final infarct.

**FIGURE 7:**
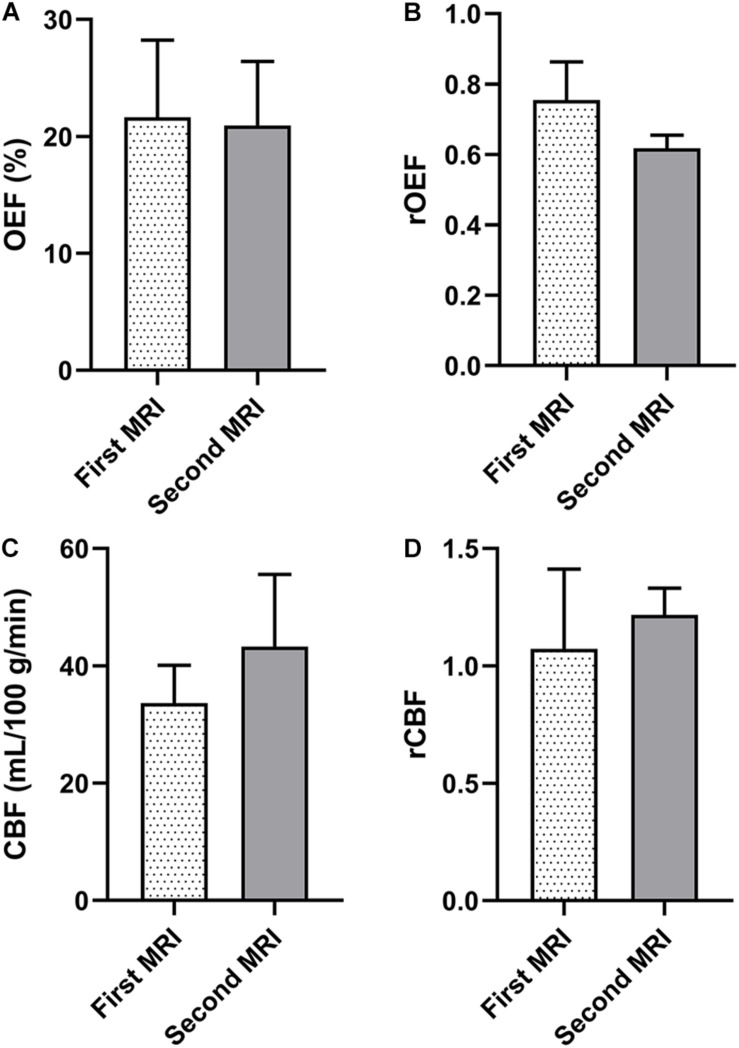
Parameters of the ischemic lesions between the longitudinal MRI scans in four patients whose IDL is of equal volume to the FI. **(A)** OEF. **(B)** rOEF. **(C)** CBF. **(D)** rCBF. OEF, oxygen extraction fraction; rOEF, relative OEF; CBF, cerebral blood flow; rCBF, relative CBF; IDL, initial diffusion lesion; FI, final infarct.

In the NDR group, the volume of the FI was larger than that of the IDL in two cases, and the enlarged part was called mismatch. The OEF, rOEF, CBF, and rCBF were decreased from the first to the second MRI in the region of the IDL, mismatch, and FI (Figure 6). Note that the rOEF of the mismatch fluctuated around 1.0 (case 6 = 0.98, case 11 = 1.04) in the first scan. In the other four cases with constant lesion volume, the OEF and rOEF decreased with time and the CBF and rCBF increased, although the difference did not reach statistical significance (Figure 7).

## Discussion

In this report, we explored the spatiotemporal evolution of OEF in ischemic stroke by means of CAT-based QQ. The OEF within the infarcted area decreased from the acute to the chronic phase amid the larger cohort. A positive association was found between OEF and the NIHSS clinical score in the acute phase when the brains were struggling to extract more oxygen and patients manifested more severe symptoms. In the smaller sub-cohort who underwent serial imaging, the OEF increased within the RR from the first MRI to the second. Enlarged lesions were also observed. The OEF of the mismatch was slightly higher than contralateral hemisphere in case 11 in the first MRI and then decreased.

The ischemic penumbra is defined as the brain tissue that received perfusion within the threshold of functional impairment and morphological integrity around the ischemic core (Astrup et al., 1981), at risk of deteriorating into ischemic and dead tissues without timely intervention or autonomous reperfusion. In this study, two patients in the NDR group showed mismatch even at the early subacute phase according to the extended FI. The OEF of the lesions was first slightly elevated and then decreased and the CBF decreased continuously. This occasional finding suggests that there still exists salvageable tissue even beyond the acute phase that would progress to infarction without suitable treatment for tissue reperfusion.

Besides the penumbra, researchers also realized that the acute restricted diffusion lesions comprise benign oligemia, which is likely to reverse (Olivot et al., 2009b; Cheung et al., 2020). The reversibility of diffusion lesions is associated with early reperfusion or recanalization of the ischemic tissue with or without endovascular therapies (Albach et al., 2013; Asdaghi et al., 2014) and absent or less severe perfusion deficit within diffusion lesions (Olivot et al., 2009a). Fiehler et al. (2004) reported ADC normalization in 14 hyperacute stroke patients, of whom nine showed complete reperfusion on MRI follow-up at day 1 and 4 showed partial reperfusion. In a more recent study (Yoo et al., 2019), complete reperfusion was found independently associated with DWI reversal after endovascular treatment. In the 11 patients with longitudinal scans in our study, five patients showed reversal portion of the IDL according to the smaller FI. At early scan times, the RR exhibited increased CBF, which is 1.0–1.5 higher than the contralateral hemisphere, and the OEF decreased accordingly for the stable CMRO_2_. Then, at later times, the CBF decreased to or below the normal level with increased OEF compared to the first scan. This spatiotemporal evolution further proves that the IDL contains penumbral tissue, which may turn into normal from the perspective of oxygen extraction. Note that the rOEF in the region of FI also increased, which could be explained by three reasons: (1) hyperintensity on T2FLAIR in 1–2 weeks of stroke is prone to including vasogenic edema, thus is not a good approximation of the FI (Gaudinski et al., 2008); (2) the tissue of the FI may be salvageable if the CBF is restored promptly; and (3) due to “stationary” deoxygenated blood without blood flow and slowed removal of deoxyhemoglobin (Stone et al., 2019).

The reversal of restricted diffusion lesions was frequently observed among cortical hyperintensities in our study and among patients of younger age and with higher CBF and minor clinical severity. These findings are in line with previous studies demonstrating DWI reversal (Labeyrie et al., 2012; Albach et al., 2013). These patients did not undergo treatment with thrombolysis or thrombectomy and were first scanned beyond 24 h from symptom onset. Although the typical clinical practice for endovascular therapy is taken within 6 h and the Endovascular Therapy Following Imaging Evaluation for Ischemic Stroke (DEFUSE 3) trial (Albers et al., 2018) extended the time window from 6 to 16 h, our study may provide evidence for reperfusion therapy in a longer time window in terms of the tissue window. Further investigations are needed to accelerate the opening of collateral circulation by a pharmacological method in patients with severe perfusion deficit and with lesions in deep white matter. Other auxiliary treatments such as modulation of neuroinflammation (Joseph et al., 2020), free radical toxicity, and apoptosis (Dirnagl et al., 1999) are also necessary to save the ischemic brain tissue and improve neurological functions.

In lacunar/subcortical ischemic stroke, the CBF decreases within the first 6 h and reaches a peak at day 7 (Lin et al., 2008; Yang et al., 2015). In the NDR group, the rOEFs of the lesions were significantly decreased from the first MRI to the second, indicating deterioration of brain tissue. Nonetheless, the rCBF values were close to 1.0 at both scans. In consideration of the lesion sites (subcortical/deep white matter), we could hypothesize that the CBF was lower at the acute phase and reached a peak at day 7, when no MRIs were taken.

QQ is a promising method integrating QSM and qBOLD that eliminates the assumption of the linear CBF/CBV in QSM and the non-blood tissue susceptibility in qBOLD. By comparing with PET in healthy adult brains, Cho et al. (2020a) reported good agreement between QQ OEF (34.2 ± 2.6%) and PET OEF (32.8 ± 6.7%). With CAT, the SNR, spatial resolution, and temporal resolution were much better than those of QQ without CAT (Cho et al., 2018). The similar values of OEF in the ischemic lesions and the similar time evolution patterns between this study and a previous one that used the same method (Zhang et al., 2020) indicate the good reproducibility of CAT-based QQ. The positive association between OEF and the NIHSS score coincides with the results in a QSM study (Fan et al., 2020). The characteristic of being vascular challenge-free makes the CAT-based QQ more convenient for clinical implementation than other hypercapnia- or hyperoxia-based MRI methods. Also, it has the advantage of exemption from radiation exposure compared to the gold standard PET.

There are some limitations in our study. CBF information for the 115 patients was removed due to incomplete perfusion imaging data. Optimization of the study design and the MRI scan protocol is warranted in future research. The sample size of patients with longitudinal scans and patients in the hyperacute phase (<6 h from stroke symptom onset) was small; thus, the difference between patients with and without DWI reversal remains unclear and the generalizability of the results is limited. Recruitment of more eligible patients is needed to validate the primary findings. Partial volume effect may have caused overestimations of the IDL and FI, leading to a risk of bias for the calculation of OEF. Further studies with smaller slice thickness of the DWI and T2 images would allow a more accurate description of OEF.

## Conclusion

In conclusion, the OEF maps generated by the CAT-based QQ method provide an improved ischemic tissue characterization and disclose selective viability of the diffusion lesions beyond the therapeutic time window, apart from the conventional penumbra, which hints at a more positive clinical treatment for the early recanalization and construction of collateral circulation.

## Data Availability Statement

The original contributions presented in the study are included in the article/Supplementary Material, further inquiries can be directed to the corresponding authors.

## Ethics Statement

The studies involving human participants were reviewed and approved by the Institutional Review Board of Tongji Hospital, Tongji Medical College, Huazhong University of Science and Technology, Wuhan, China. The ethics committee waived the requirement of written informed consent for participation.

## Author Contributions

DW conceptualized the study, performed the investigation and formal analysis, curated the data, wrote the original draft, and helped with the visualization. YZ, NS, SL, YQ, and GZ performed the validation, investigation, and formal analysis, and curated the data. SY and YX contributed to the validation and investigation, and curated the data. SZ and WZ conceptualized the study, review and edited the manuscript, and helped with the supervision, project administration, and funding acquisition. JC and YW helped with the methodology and software. All authors have read the final version of the manuscript and approved it for publication.

## Conflict of Interest

The authors declare that the research was conducted in the absence of any commercial or financial relationships that could be construed as a potential conflict of interest.

## Publisher’s Note

All claims expressed in this article are solely those of the authors and do not necessarily represent those of their affiliated organizations, or those of the publisher, the editors and the reviewers. Any product that may be evaluated in this article, or claim that may be made by its manufacturer, is not guaranteed or endorsed by the publisher.

## Footnotes

1
www.itksnap.org/

## Abbreviations

CAT: cluster analysis of time evolution
FI: final infarct
IDL: initial diffusion lesion
DR group: group with regional diffusion reversal
NDR group: group without diffusion reversal
QQ: QSM + qBOLD
rCBF: relative cerebral blood flow
rOEF: relative oxygen extraction fraction
RR: reversal region.
