# Supplementary material for: The Spatiotemporal Evolution of MRI-Derived Oxygen Extraction Fraction and Perfusion in Ischemic Stroke
Source: Front Neurosci. 2021 Aug 16;15:716031. doi: 10.3389/fnins.2021.716031 (PMC8415351; doi:10.3389/fnins.2021.716031)
Supplement: Supplementary file 1 [file Table_1.DOCX]

Supplementary Material

# Supplementary Tables

| Supplementary Table 1. Patient characteristics of the 11 ischemic stroke patients with longitudinal MRI scans. | | | | | | | | | | | | |
| --- | --- | --- | --- | --- | --- | --- | --- | --- | --- | --- | --- | --- |
| Patient | Sex | Age | Side, symptom | Time to first MRI (h) | Time to second MRI (d) | Offending vessel | Baseline NIHSS | NIHSS at second MRI | Initial diffusion lesion volume (ml) | Final infarct volume (ml) | Lesion reversibility rate | TOAST criteria |
| 1 | M | 52 | R, limb weakness | 72 | 13 | L-MCA, M2 | 8 | 6 | 2.49 | 2.49 | 0 | large-artery atherosclerosis |
| 2 | M | 59 | L, limb weakness | 50 | 8 | R-MCA, M2 | 2 | 1 | 2.82 | 2.82 | 0 | large-artery atherosclerosis |
| 3 | M | 57 | R, limb weakness | 58 | 9 | L-MCA, M2 | 2 | 2 | 13.18 | 8.3 | 37.03% | large-artery atherosclerosis |
| 4 | M | 35 | R, upper limb weakness, aphasia | 56.5 | 12 | L-ICA | 6 | 1 | 40.08 | 13.88 | 65.37% | large-artery atherosclerosis |
| 5 | M | 54 | L, limb weakness | 36 | 10 | R-MCA | 2 | 1 | 1.56 | 1.56 | 0 | Small-artery occlusion |
| Supplementary Table 1. Continued. | | | | | | | | | | | | |
| Patient | Sex | Age | Side, symptom | Time to first MRI (h) | Time to last MRI scan(d) | Offending vessel | Baseline NIHSS | NIHSS at second MRI | Initial diffusion lesion volume (ml) | Final infarct volume (ml) | Lesion reversibility rate | TOAST criteria |
| 6 | M | 57 | R, limb weakness, aphasia | 72 | 10 | L-MCA, M1 | 6 | 6 | 7.76 | 20.97 | -170.23% | large-artery atherosclerosis |
| 7 | M | 52 | L, limb weakness | 64 | 12 | R-ICA | 5 | 4 | 5.71 | 5.71 | 0 | large-artery atherosclerosis |
| 8 | F | 33 | Aphasia | 96 | 8 | L-MCA, M1 | 6 | 1 | 25.45 | 12.73 | 49.98% | large-artery atherosclerosis |
| 9 | M | 46 | L, limb weakness | 84 | 40 | R-MCA, M1 | 1 | 0 | 2.71 | 1.64 | 39.48% | large-artery atherosclerosis |
| 10 | M | 56 | R, limb weakness and numbness | 74 | 20 | NA | 2 | 1 | 1.1 | 0.18 | 83.64% | Undetermined etiology |
| 11 | F | 64 | R, limb weakness, aphasia | 37.5 | 10 | L-MCA, M1 | 8 | 11 | 5.31 | 10.97 | -106.59% | large-artery atherosclerosis |
| TOAST, Trial of ORG 10172 in Acute Stroke Treatment; L, left; R, right; ICA, internal carotid artery; MCA, middle cerebral artery; NA, not applicable. | | | | | | | | | | | | |
